# Microparticle Acoustophoresis in Aluminum-Based Acoustofluidic Devices with PDMS Covers

**DOI:** 10.3390/mi11030292

**Published:** 2020-03-11

**Authors:** William Naundrup Bodé, Lei Jiang, Thomas Laurell, Henrik Bruus

**Affiliations:** 1 Department of Physics, Technical University of Denmark, DTU Physics Building 309, DK-2800 Kongens Lyngby, Denmark; 2 Department of Biomedical Engineering, Lund University, 221 00 Lund, Sweden; jiang.lei@bme.lth.se (L.J.); thomas.laurell@bme.lth.se (T.L.)

**Keywords:** acoustofluidics, microparticle acoustophoresis, numerical modeling, aluminum microdevices, polydimethylsiloxane (PDMS) covers

## Abstract

We present a numerical model for the recently introduced simple and inexpensive micromachined aluminum devices with a polydimethylsiloxane (PDMS) cover for microparticle acoustophoresis. We validate the model experimentally for a basic design, where a microchannel is milled into the surface of an aluminum substrate, sealed with a PDMS cover, and driven at MHz frequencies by a piezoelectric lead-zirconate-titanate (PZT) transducer. Both experimentally and numerically we find that the soft PDMS cover suppresses the Rayleigh streaming rolls in the bulk. However, due to the low transverse speed of sound in PDMS, such devices are prone to exhibit acoustic streaming vortices in the corners with a relatively large velocity. We predict numerically that in devices, where the microchannel is milled all the way through the aluminum substrate and sealed with a PDMS cover on both the top and bottom, the Rayleigh streaming is suppressed in the bulk thus enabling focusing of sub-micrometer-sized particles.

## 1. Introduction

Acoustofluidic devices based on bulk acoustic waves for microparticle handling are traditionally made by microfabrication in acoustically hard materials, such as silicon or glass, yielding acoustic resonators with relatively high Q values [1]. Such devices can be fabricated controllable and with high accuracy, but may suffer from a high production cost. Several solutions have been proposed and successfully demonstrated to achieve simple and inexpensive acoustofluidic devices. Simple mass-produced glass capillary tubes have been used for trapping of microparticles [2,3,4,5,6,7] and even nanoparticles [8,9]. The first attempts of using polymer-based devices have been published showing applications such as focusing of polymer beads [10,11,12,13], lipids [10], and red blood cells [11,14], as well as blood-bacteria separation [15] and purification of lymphocytes [16]. Although a cheaper material, polymers are difficult to use in acoustofluidics due to their low acoustic contrast relative to water, but recently it was shown how to circumvent this problem by use of the whole-system-resonance principle [17]. According to this principle, the acoustic contrast between the ambient air and the whole device allows the excitation of specific whole-system vibrational resonance modes, which support strong acoustofluidic responses in a given embedded liquid-filled cavity, without the corresponding acoustic pressure being a localized cavity resonance as in conventional devices.

**Figure 1:**
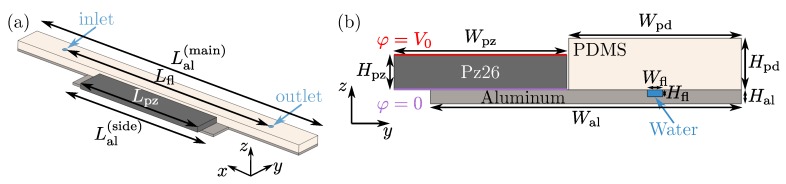
(**a**) 3D sketch drawn to scale of the single-PDMS-cover (beige) aluminum-based (light gray) device driven by a piezoelectric PZT-Pz26 transducer (dark gray, placed on a protrusion next to the PDMS) with silver electrodes (not shown). The inlet and outlet channels are marked by small circles. (**b**) Cross-sectional view of the device at the vertical center plane, used in the 2D model presented in Section 3, where the 9-µm-thick electrodes of the Pz26 transducer are connected to ground (purple) and to the driving ac-voltage (red). The gap between the Pz26 and the PDMS cover is $\frac{1}{8}W_{\mathrm{fl}}$.

Another solution is to combine acoustically hard materials with the soft rubber polydimethylsiloxane (PDMS). In 2012, Adams et al., presented experiments and simulations of a high-throughput, temperature- controlled microchannel acoustophoresis device made by rapid prototyping. The device was based on a PDMS-gasket defining the side-walls and shape of the acoustofluidic chamber, which then was sealed using standard microscope slides [18]. Also Xu et al., used such a glass-PDMS-glass structure in their recent device for isolation of cells from dilute samples using bead-assisted acoustic trapping [19]. Similarly in 2018, Gautam et al., designed, fabricated, and tested simple and inexpensive micromachined PDMS-covered aluminum-based microfluidic devices for acoustic focusing of particles and cells [20]. These devices appear to be versatile and truly simple to fabricate, as the desired microchannel system is micromilled into the surface of an aluminum base and bonded with a PDMS cover. Since neither theory nor simulation was presented by Gautam et al. [20], we develop in this work a numerical model for such devices and validate them experimentally: In Section 3 for a basic design similar to that in Ref. [20], where a microchannel is micromilled into the surface of an aluminum base and sealed with a PDMS cover as sketched in Figure 1 and with the dimensions given in Table 1; and in Section 4 for a geometrically symmetric, but anti-symmetrically ac-voltage-actuated device.

In the main part of our work, we use the validated numerical model to predict the answer to the following three questions: (1) Does the exceptionally low transverse speed of sound ∼100 m/s in the nearly incompressible PDMS imply a singular behavior of the acoustic streaming near the PDMS-aluminum corners of the device? (2) Does anti-symmetric excitation using a split-top-electrode configuration as in Ref. [17] lead to better acoustophoresis? And finally, (3) does the use of the acoustically soft PDMS cover lead to a suppression of the bulk-streaming rolls generated by the water-PDMS interface, and if so, would PDMS covers sealing both the top and bottom part of devices, where the microchannel is milled all the way through the aluminum base, lead to a suppression of all bulk-streaming rolls in the device? The answers to these questions turn out to be predominantly affirmative.

## 2. Theory: The Governing Equations and Boundary Conditions

We follow the work by Skov et al. [21] in our theoretical and numerical modeling of the acoustofluidic system sketched in Figure 1. The continuum fields of the model are the following: An electric potential scalar field $\tilde{\varphi }\left(r,t\right)$ is present in the piezoelectric domain (Pz26), an elastic displacement vector field $\tilde{u}\left(r,t\right)$ is present in all four solid domains (Pz26, aluminum, silver electrodes, and PDMS), and an acoustic pressure scalar field ${\tilde{p}}_{1}\left(r,t\right)$ as well as a steady acoustic streaming velocity vector field $v_{2}\left(r\right)$ and pressure $p_{2}\left(r\right)$ are present in the water-filled microchannel. As in typical experiments, the system is driven by a time-harmonic ac-voltage $V_{0}\;e^{-i\omega t}$ of amplitude $V_{0}$ and angular frequency ω=2πf, where *f* is the ultrasound frequency, applied to the silver electrodes of the Pz26 transducer. Consequently, to first order in the applied voltage, all fields acquire a time-harmonic phase factor $e^{-i\omega t}$ and a complex-valued spatially varying amplitude,

(1) $$\tilde{\varphi }\left(r,t\right)=\varphi \left(r\right)e^{-i\omega t},\;\tilde{u}\left(r,t\right)=u\left(r\right)e^{-i\omega t},\;\mathrm{and}\;{\tilde{p}}_{1}\left(r,t\right)=p_{1}\left(r\right)e^{-i\omega t}.$$

In the following, the common phase factor $e^{-i\omega t}$ is left out of the linear equations. Only the space-dependent amplitudes are computed, but multiplying them by $e^{-i\omega t}$ recovers the time dependence.

### 2.1. The Piezoelectric Transducer

We model a standard lead-zirconate-titanate (PZT) transducer of type Pz26 polarized in the *z* direction. The mechanical stress tensor σ and the electric displacement field D are given in terms of the gradients of the elastic displacement field u and the electric potential φ by the electromechanical coupling matrix. Using the compact Voigt notation, this constitutive relation is given by

(2) $$\left(\begin{array}{c} \sigma_{xx}\\ \sigma_{yy}\\ \sigma_{zz}\\ \sigma_{yz}\\ \sigma_{xz}\\ \sigma_{xy}\\ D_{x}\\ D_{y}\\ D_{z} \end{array}\right)=\left(\begin{array}{ccccccccc} C_{11} & C_{12} & C_{13} & 0 & 0 & 0 & 0 & 0 & -e_{31}\\ C_{12} & C_{11} & C_{13} & 0 & 0 & 0 & 0 & 0 & -e_{31}\\ C_{13} & C_{13} & C_{33} & 0 & 0 & 0 & 0 & 0 & -e_{33}\\ 0 & 0 & 0 & C_{44} & 0 & 0 & 0 & -e_{15} & 0\\ 0 & 0 & 0 & 0 & C_{44} & 0 & -e_{15} & 0 & 0\\ 0 & 0 & 0 & 0 & 0 & C_{66} & 0 & 0 & 0\\ 0 & 0 & 0 & 0 & e_{15} & 0 & \varepsilon_{11} & 0 & 0\\ 0 & 0 & 0 & e_{15} & 0 & 0 & 0 & \varepsilon_{11} & 0\\ e_{31} & e_{31} & e_{33} & 0 & 0 & 0 & 0 & 0 & \varepsilon_{33} \end{array}\right)\;\left(\begin{array}{c} \partial_{x}\;u_{x}\\ \partial_{y}\;u_{y}\\ \partial_{z}\;u_{z}\\ \partial_{y}u_{z}+\partial_{z}\;u_{y}\\ \partial_{x}u_{z}+\partial_{z}\;u_{x}\\ \partial_{x}u_{y}+\partial_{y}\;u_{x}\\ -\partial_{x}\varphi \\ -\partial_{y}\varphi \\ -\partial_{z}\varphi \end{array}\right).$$

Here, $C_{ik}$ are the elastic coefficients, $\varepsilon_{ik}$ are the electric permittivities, and $e_{ik}$ are the piezoelectric coupling constants. The remaining components of σ are given by the symmetry relations $\sigma_{ik}=\sigma_{ki}$.

The governing equations in the piezoelectric Pz26 material of mass density $\rho_{\mathrm{sl}}$ are the Cauchy equation for the elastic displacement field u and, for frequencies less than 100 MHz in systems smaller than 1 m without free charges, the quasi-static Gauss law for the electric potential φ,

(3) $$-\omega^{2}\rho_{\mathrm{sl}}u=\nabla \cdot \sigma ,\;\nabla \cdot D=0.$$

The boundary conditions for the Pz26 domain with surface normal vector n are continuity of φ, u and σ·n across the elastic solid interfaces to the silver electrodes, and zero stress as well as zero electric charge on surfaces exposed to air,

(4a) $$\begin{array}{cccc} \mathrm{Pz}26\;\mathrm{domain} & \leftarrow \mathrm{charged}\;\mathrm{electrode}:\; & \varphi & =V_{0},\;\sigma \cdot n\;\mathrm{and}\;u\;\mathrm{continuous}, \end{array}$$

(4b) $$\begin{array}{cccc} \mathrm{Pz}26\;\mathrm{domain} & \leftarrow \mathrm{grounded}\;\mathrm{electrode}:\; & \varphi & =0,\;\sigma \cdot n\;\mathrm{and}\;u\;\mathrm{continuous}, \end{array}$$

(4c) $$\begin{array}{cccc} \mathrm{Pz}26\;\mathrm{domain} & \leftarrow \mathrm{air}: & \;n\cdot D & =0\;\mathrm{and}\;\sigma \cdot n=0. \end{array}$$

Here, the notation A←B refers to the influence of *B* on domain *A* with outward surface normal n.

### 2.2. The Elastic Aluminum Base, PDMS Cover, and Silver Electrodes

The aluminum base, the PDMS cover, and the silver electrodes on the Pz26 transducer can all be described as isotropic, linear elastic materials. Again using the compact Voigt notation, the corresponding constitutive equation relating the strain and the stress are given by

(5) $$\left(\begin{array}{c} \sigma_{xx}\\ \sigma_{yy}\\ \sigma_{zz}\\ \sigma_{yz}\\ \sigma_{xz}\\ \sigma_{xy} \end{array}\right)=\left(\begin{array}{cccccc} C_{11} & C_{12} & C_{12} & 0 & 0 & 0\\ C_{12} & C_{11} & C_{12} & 0 & 0 & 0\\ C_{12} & C_{12} & C_{11} & 0 & 0 & 0\\ 0 & 0 & 0 & C_{44} & 0 & 0\\ 0 & 0 & 0 & 0 & C_{44} & 0\\ 0 & 0 & 0 & 0 & 0 & C_{44} \end{array}\right)\;\left(\begin{array}{c} \partial_{x}\;u_{x}\\ \partial_{y}\;u_{y}\\ \partial_{z}\;u_{z}\\ \partial_{y}u_{z}+\partial_{z}\;u_{y}\\ \partial_{x}u_{z}+\partial_{z}\;u_{x}\\ \partial_{x}u_{y}+\partial_{y}u_{x} \end{array}\right).$$

For isotropic materials only $C_{11}$ and $C_{44}$ are independent, since the constraint $C_{12}=C_{11}-2C_{44}$ applies. The transverse and longitudinal speed of sound becomes $c_{\mathrm{lo}}=\sqrt{C_{11}/\rho_{\mathrm{sl}}}$ and $c_{\mathrm{tr}}=\sqrt{C_{44}/\rho_{\mathrm{sl}}}$, respectively. The governing equation for the displacement field u in an elastic solid with mass density $\rho_{\mathrm{sl}}$ is the Cauchy equation

(6) $$-\omega^{2}\rho_{\mathrm{sl}}u=\nabla \cdot \sigma .$$

The boundary conditions for the elastic solid domains with surface normal vector n are continuity of u and σ·n across the elastic solid interfaces, and zero stress on surfaces exposed to air,

(7a) $$\begin{array}{ccc} \mathrm{solid}\;\mathrm{domain} & \leftarrow \mathrm{adjacent}\;\mathrm{solid}:\; & \sigma \cdot n\;\mathrm{and}\;u\;\mathrm{continuous}, \end{array}$$

(7b) $$\begin{array}{ccc} \mathrm{solid}\;\mathrm{domain} & \leftarrow \mathrm{air}: & \sigma \cdot n=0. \end{array}$$

The boundary conditions for the fluid-solid interfaces are given below.

### 2.3. Pressure Acoustics in the Water-Filled Microchannel

The governing equation for the acoustic pressure $p_{1}$ inside a fluid with mass density $\rho_{\mathrm{fl}}$, speed of sound $c_{\mathrm{fl}}$, dynamic viscosity $\eta_{\mathrm{fl}}$, and bulk viscosity $\eta_{\mathrm{fl}}^{b}$ is the Helmholtz equation

(8) $$\nabla^{2}p_{1}+\frac{\omega^{2}}{c_{\mathrm{fl}}^{2}}\left(1+i\frac{\Gamma_{\mathrm{fl}}}{2}\right)p_{1}=0,\;\mathrm{where}\;\Gamma_{\mathrm{fl}}=\left(\frac{4}{3}\eta_{\mathrm{fl}}+\eta_{\mathrm{fl}}^{b}\right)\omega \kappa_{\mathrm{fl}}\;\mathrm{and}\;\kappa_{\mathrm{fl}}=\frac{1}{\rho_{\mathrm{fl}}c_{\mathrm{fl}}^{2}}.$$

The acoustic velocity $v_{1}$ in the bulk of the fluid is a potential flow given by the acoustic pressure $p_{1}$,

(9) $$v_{1}=-i\frac{1-i\Gamma_{\mathrm{fl}}}{\omega \rho_{\mathrm{fl}}}\nabla p_{1}.$$

The boundary conditions for the fluid-solid interface are continuity of the stress and the velocity. In Ref. [21] the explicit form of these boundary conditions were derived taking into account the viscous boundary layer in the fluid. By introducing the shear wavenumber $k_{s}=\left(1+i\right)/\delta$, where $\delta =\sqrt{2\eta_{\mathrm{fl}}/\left(\omega \rho_{\mathrm{fl}}\right)}$ is the thickness of the viscous boundary layer, and expressing the displacement velocity as $v_{\mathrm{sl}}=-i\omega u$, the fluid-solid boundary conditions become,

(10a) $$\begin{array}{cccc} \mathrm{Solid}\;\mathrm{domain} & \leftarrow \mathrm{fluid}:\; & \sigma \cdot n & =-p_{1}n+ik_{s}\eta_{\mathrm{fl}}(v_{\mathrm{sl}}-v_{1}), \end{array}$$

(10b) $$\begin{array}{cccc} \mathrm{Fluid}\;\mathrm{domain} & \leftarrow \mathrm{solid}: & v_{1}\cdot n & =v_{\mathrm{sl}}\cdot n+\frac{i}{k_{s}}\nabla_{\|}\cdot {\left(v_{\mathrm{sl}}-v_{1}\right)}_{\|}. \end{array}$$

Here, the viscous loss in the fluid is taken into account in the boundary conditions, and it appears through the slip velocity $v^{\delta }=\left(v_{\mathrm{sl}}-v_{1}\right)$.

### 2.4. Acoustic Streaming in the Water-Filled Microchannel

Steady acoustic streaming arises in the fluid due to the inherent nonlinear fluid dynamics, here the time-average of products of time-harmonic fields. Following Bach and Bruus [22], the streaming velocity $v_{2}$ is governed by a real-valued incompressible Navier–Stokes equation with a body force due to the real part of the acoustic energy-flux density,

(11) ∇·v2=0and0=−∇p2+ηfl∇2v2+Γflω2cfl2Re{p1*v1}.

The no-slip condition on the fluid-solid interface requires that $v_{2}=-\langle \left(u\cdot \nabla \right)v_{1}\rangle$, which leads to the boundary condition for the acoustic streaming $v_{2}$ at the fluid-solid interface,

(12a) $$\begin{array}{cc} v_{2} & =\left(A\cdot e_{\|}\right)\;e_{\|}+\left(B\cdot e_{⊥}\right)\;e_{⊥}, \end{array}$$

(12b) A=−12ωRe{v1δ0*·∇12v1δ0−ivsl0−ivsl0*·∇v1+2−i2∇·v1δ0*+i∇·vsl0*−∂⊥v1·e⊥*v1δ0},

(12c) B=12ωRe{v10*·∇v1},

where the superscript “0” indicates a field evaluated along the fluid-solid interface. The subscripts ‖ and ⊥ and the unit vectors $e_{\|}$ and $e_{⊥}$ refer to the parallel and normal direction to the solid wall respectively. The steady pressure $p_{2}$ accompanying $v_{2}$ is governed by the continuity for $v_{2}$, and since it only appears as a gradient in Equation (11), we must fix the level of $p_{2}$ by the constraint $\int_{\Omega_{\mathrm{fl}}}p_{2}\;dydz=0$.

### 2.5. The Acoustic Radiation and Drag Force on Suspended Microparticles

Suspended spherical particles of radius *a* and mass density $\rho_{\mathrm{pa}}$ will experience the radiation force $F_{\mathrm{rad}}$ due to acoustic scattering, which is given by the potential $U_{\mathrm{rad}}$ with monopole and dipole scattering coefficients $f_{0}$ and $f_{1}$, respectively [23],

(13) $$F_{\mathrm{rad}}=-\nabla U_{\mathrm{rad}}\;\mathrm{where}\;U_{\mathrm{rad}}=\frac{4}{3}\pi a^{3}\left(\frac{1}{4}f_{0}\kappa_{\mathrm{fl}}|p_{1}{|}^{2}-\frac{3}{8}f_{1}\rho_{\mathrm{fl}}{\left|v_{1}\right|}^{2}\right).$$

The velocity $v_{\mathrm{pa}}$ of a suspended particle at position r(t) in the water is determined by a balance between the Stokes drag force $F_{\mathrm{drag}}=6\pi \eta_{\mathrm{fl}}a\left(v_{2}-v_{\mathrm{pa}}\right)$, the acoustic radiation force $F_{\mathrm{rad}}$, and the buoyancy force $F_{\mathrm{buoy}}=-\frac{4}{3}\pi a^{3}\left(\rho_{\mathrm{pa}}-\rho_{\mathrm{fl}}\right)ge_{z}$,

(14) $$v_{\mathrm{pa}}\left(r\right)=v_{2}\left(r\right)+\frac{F_{\mathrm{rad}}\left(r\right)}{6\pi \eta_{\mathrm{fl}}a}-\frac{2}{9}\frac{a^{2}}{\eta_{\mathrm{fl}}}\left(\rho_{\mathrm{pa}}-\rho_{\mathrm{fl}}\right)ge_{z}.$$

The particle trajectory is then given by integration in time as $r_{\mathrm{pa}}\left(t\right)=\int_{0}^{t}v_{\mathrm{pa}}\left(r(t^{\prime })\right)\;dt^{\prime }$. Inertia is neglected here, because the largest particle with radius a=2.4µm moves slower than $v_{\mathrm{pa}}<1$ cm/s yielding a small particle Reynolds number, Repa=1ηflρflavpa≈0.03≪1.

We define the horizontal *y* axis such that the channel is centered around y=0. To determine at which frequencies *f* acoustic resonance modes appear that leads to good particle focusing towards the vertical center plane located at y=0, we introduce the focusing figure of merit F(f). This is a modified version of the figure of merit R defined in Ref. [17], as follows: F should be large, when at the same time the average acoustic energy density $E_{\mathrm{ac}}$ is large, and the acoustic radiation force $F_{\mathrm{rad}}$ has the property that its average horizontal *y* component is large and points toward the center, whereas its average vertical *z* component has a small magnitude,

(15) $$F\left(f\right)=\frac{\int_{\Omega_{\mathrm{fl}}}\mathrm{sgn}\left(-y\right)\;F_{\mathrm{rad},y}\;dydz}{\int_{\Omega_{\mathrm{fl}}}\left|F_{\mathrm{rad},z}\right|\;dydz}\;E_{\mathrm{ac}},\;\mathrm{where}\;E_{\mathrm{ac}}=\frac{\int_{\Omega_{\mathrm{fl}}}\left[\frac{1}{4}\kappa_{\mathrm{fl}}|p_{1}{|}^{2}+\frac{1}{4}\rho_{\mathrm{fl}}{\left|v_{1}\right|}^{2}\right]\;dydz}{\int_{\Omega_{\mathrm{fl}}}1\;dydz}.$$

Here, sgn(−y) designates minus the sign of the *y* coordinate.

## 3. Numerical Implementation and Experimental Validation of the Single-PDMS-Cover Model

To illustrate the numerical implementation of the model, we choose the geometry as sketched in Figure 1, with the dimensions listed in Table 1, and the material parameters given in Appendix A. This device geometry is similar to the one in Ref. [20], namely an aluminum-based device with a straight channel and a single PDMS cover. Our general numerical modeling is then validated experimentally for this device and further supported by the results presented in Section 4, for the anti-symmetric actuated design.

### 3.1. Model Implementation in COMSOL Multiphysics

The model system of Figure 1 as well as its governing equations and boundary conditions given in Section 2, are implemented in the commercial finite-element method software COMSOL Multiphysics 5.4 [24] using the *weak form PDE* module, closely following the method presented in Ref. [21]. We use quartic Lagrange shape functions for $p_{1}$, cubic for u, φ, and $v_{2}$, and quadratic for $p_{2}$. For simplicity, we approximate the device by an infinitely long straight channel, and thus restrict the computation to the 2D cross section. The material parameters used in the model are given in Appendix A. The simulations were performed on a workstation with a 3.5-GHz Intel Xeon CPU E5-1650 v2 dual-core processor and with 128 GB RAM.

### 3.2. Manufacturing Method of the Chip for the Experimental Validation

**Figure 2:**
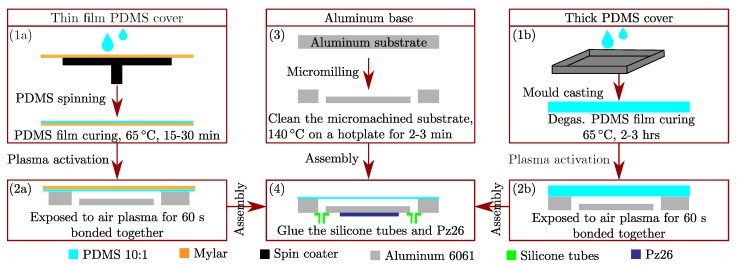
Schematic showing the sequence in the fabrication of the aluminum devices with PDMS covers. Production of a thin PDMS cover on a Mylar sheet by (**1a**) spinning and curing followed by (**2a**) bonding to the aluminum base by plasma activation and removal of the Mylar sheet. (**1b**) Production of a thick PDMS cover by mould casting followed by (**2b**) bonding to the aluminum base by plasma activation. (**3**) Fabrication of the channel in the aluminum base by micromilling. (**4**) Attachment of silicone tubes and the Pz26 transducer.

A sketch of the device fabrication method is shown in Figure 2. A more detailed description of the process steps is given in the following.

*The aluminum base*. A microchannel was milled in an aluminum substrate (alloy 6061, McMaster-Carr, Los Angeles, CA, USA) using a CNC milling machine (Solectro AB, Lomma, Sweden). The micromachined substrate was cleaned with acetone, ethanol, and Milli-Q (Millipore Corporation, Burlington, MA, USA), and dried on a 140 ${}^{\circ }$C hotplate for 2–3 min prior to bonding with the PDMS film covering the channel. This constitutes the base of the device.

*The PDMS covers*. Sylgard 184 silicone elastomer (Dow Corning, Ellsworth Adhesives, Germantown, WI, USA) was mixed with the curing agent at the commonly used weight ratio of 10:1 and degassed. Two types of covers were made: (a) Thin PDMS-film covers, by deposition of 1 mL of elastomer on a 100-µm-thick 100-mm-by-100-mm plastic transparency sheet (Mylar), followed by spin-coating and curing at 65 ${}^{\circ }$C for 15–30 min, and (b) 1.5-mm-thick PDMS covers by conventional mould casting.

*Device assembly*. Afterwards, the cured PDMS film and cleaned aluminum substrates were treated with air plasma in a Zepto plasma cleaner (Diener electronic GmbH + Co. KG, Ebhausen, Germany) for 60 s. PDMS and aluminum were subsequently bonded together and cured at 80 ${}^{\circ }$C for 4 min. After curing, the Mylar sheet was removed from the PDMS-aluminum assembly. For flow connections, silicone tubes with inner diameters that match 1/16” Teflon tubings, were glued to the base of the device. A PZT ceramic transducer (Pz26, Meggitt A/S, Kvistgaard, Denmark) designed for 2 MHz actuation was superglued to the final device.

### 3.3. Experimental Validation of the Numerical Model

The electrodes of the Pz26 transducer were coupled to an ac-voltage generator operating at 20 V peak-to-peak ($V_{0}=10$ V) at frequencies ranging from 1.5 to 2.5 MHz. After stopping the particle-loading flow, the position and velocity $v_{\mathrm{pa}}$ of fluorescently-marked polystyrene particles (see Table A3 in Appendix A) was measured using the single-camera general defocusing particle tracking (GDPT) technique [25,26] with fluorescent polystyrene beads. We use a 10µm×5µm grid size and a recorded image frame rate of 10 Hz, and the motion of 2a=4.8, 2.0, and 1.0µm-diameter tracer particles is tracked for 30, 60, and 120 s, respectively. During the data processing, the outliers were filtered out by limiting the displacement deviation of all the particles, by limiting the velocity magnitude, and by restricting the particle count to 2 in each grid.

**Figure 3:**
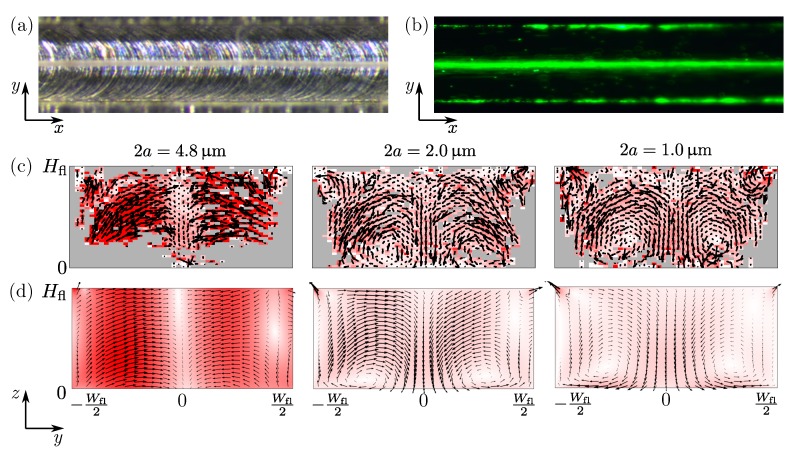
(**a**) Bright-field micrograph under flow-through condition showing acoustophoretic focusing of 4.8-µm-diameter polystyrene particles at the center (gray band). (**b**) Similarly for fluorescently-marked particles, but now also revealing that some particles are accumulating at the upper corners of the channel. (**c**) Vector plot (black arrows) and color plot ranging from 0µm/s (white) up to 40µm/s (red) of the particle velocities $v_{\mathrm{pa}}$ in the vertical cross section measured by GDPT at resonance mode “S”, f=2.048MHz, for different particle diameter 2a=4.8µm, 2.0µm, and 1.0µm. (**d**) Same vector and color plot as (**c**) but for the simulated results of $v_{\mathrm{pa}}$ in the 2D geometry shown in Figure 1b at the numerically determined resonance frequency f=1.803MHz (12% lower).

To validate our 2D model, we compare in Figure 3 simulated and measured particle behavior. The top row show top-view micrographs of the microchannel under flow-through condition, where 4.8-µm-diameter particles in bright-field are seen to focus in the center, Figure 3a. This resonance mode is called “S” for “side actuated” and was located at f=2.048MHz. This focusing is confirmed by the fluorescent image Figure 3b, which however also reveals particles accumulating near the corners of the device. In Figure 3c,d, we show measured and simulated particle velocities $v_{\mathrm{pa}}$ in the vertical cross section for different particle diameters. It is seen that the 2D model captures the following five main features in the measured particle velocity field, even though the 2D model geometry of Figure 1b assumes a translational invariant cross section along the *x* direction, which the experimental 3D geometry of Figure 1a clearly does not have. (1) The numerically predicted resonance is located at f=1.803MHz, only 12% below the experimental value. (2) As expected, for large particles 2a=4.8µm the motion is dominated by the radiation force, which is partly focusing in the vertical nodal plane at y=0 and partly pointing towards the soft PDMS cover and the top corners. As the particle size is reduced to 2a=2.0µm and then further to 2a=1.0µm, acoustic streaming becomes more dominant for the particle motion. This cross-over is clearly seen both in model and measurement of Figure 3c,d in the form of streaming flow rolls. (3) Near the hard aluminum bottom, the classical pair of counter-rotating Rayleigh streaming rolls appears. (4) Near the soft PDMS cover, the acoustic streaming rolls are confined to the top corners where it reaches its maximum value. This is a consequence of the low transverse speed of sound ∼100 m/s in PDMS, or equivalently, the nearly incompressible nature of PDMS. Finally, (5) The magnitude $v_{\mathrm{pa}}=40\;µm/s$ of the simulated particle velocity matches that of the measured one for the 4.8-µm-diameter particles, whereas it is roughly 30–50% smaller for the 2.0- and 1.0-µm-diameter particles.

## 4. Modeling of Single-PDMS-Cover Devices with Anti-Symmetric Voltage Actuation

**Figure 4:**
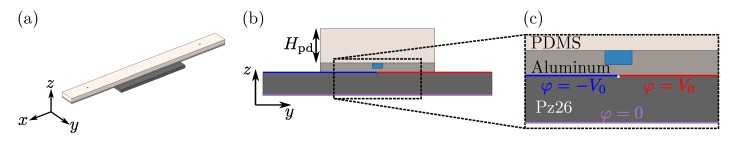
(**a**) 3D sketch drawn to scale of the proposed device driven by an anti-symmetric ac voltage. (**b**) The vertical cross section showing the thickness $H_{\mathrm{pd}}$ of the PDMS cover. (**c**) Zoom-in on the water-filled microchannel showing the PDMS cover, the aluminum base, the Pz26 transducer, and its 9-µm-thick silver electrodes, with ground on the bottom electrode (purple) and anti-symmetric ac voltage positive/negative (blue/red) applied to the split top electrode.

Following the results of Ref. [17], we investigate numerically, if better acoustophoresis, quantified by the focusing figure of merit F defined in Equation (15), is obtained by exciting the half-wave resonance mode (which is anti-symmetric around the nodal plane at y=0). This is achieved by splitting the top electrode and applying an anti-symmetric ac voltage, as sketched in Figure 4 for a device with a split-gap of size 50µm×40µm cut into the top surface of the Pz26 transducer. Here, the PDMS, the channel, and the aluminum base are translational invariant along the *x* direction.

### 4.1. Numerical Optimization of the Thickness of the PDMS Cover

**Figure 5:**
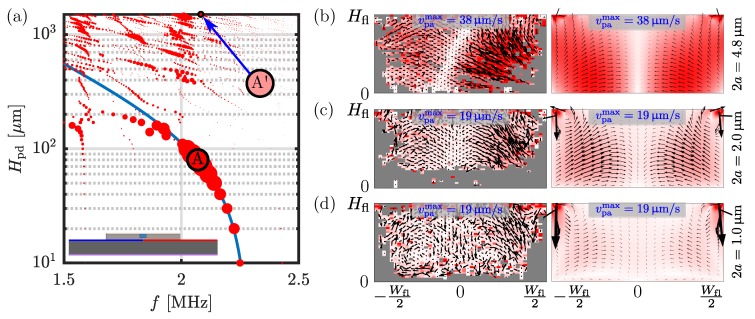
(**a**) Resonance peaks in the single-PDMS-cover aluminum-based device with anti-symmetric voltage actuation, Figure 4, as a function of the actuation frequency *f* and the thickness $H_{\mathrm{pd}}$ of the PDMS cover. The area of the points is proportional to the focusing figure of merit F defined in Equation (15). The “A${}^{\prime }$” marks the resonance mode for $H_{\mathrm{pd}}=1.5$ mm as in mode “S” of Figure 3. The “A” marks the resonance for the optimal cover thickness $H_{\mathrm{pd}}=80\;µm$. The blue curve indicates resonances sensitive to $H_{\mathrm{pd}}$. (**b**–**d**) Color and vector plot of the measured (left column) and simulated (right column) acoustophoretic particle velocity $v_{\mathrm{pa}}$ of mode “A${}^{\prime }$”, for particle diameters 4.8, 2.0, and 1.0 µm, respectively. The measured resonance frequency was f=2.052MHz and the simulated was found at f=2.095MHz (2% higher). All color plots range from 0 µm/s (white) up to $v_{\mathrm{pa}}^{\max}$ (red) with values 38 or 19 µm/s as indicated in each panel.

To illustrate how the thickness $H_{\mathrm{pd}}$ of the PDMS cover affects the resonances, we vary $H_{\mathrm{pd}}$, and for each value, we sweep the frequency *f* from 1.5 MHz to 2.5 MHz in steps of 5 kHz to locate the resonances in terms of peaks in the focusing figure of merit F versus *f*. Such a spectrum is plotted in Figure 5a, where the area of each point is proportional to F. For the same cover thickness $H_{\mathrm{pd}}=1.5$ mm as in the side-actuated mode “S” of Figure 3 with $E_{\mathrm{ac}}=8.9\;J/m^{3}$, we find a resonance mode “A${}^{\prime }$” at f=2.095 MHz with $E_{\mathrm{ac}}=4.6\;J/m^{3}$. An even better resonance mode can be found. In Figure 5a a strong resonance, which depends on the PDMS-cover thickness, is indicated by the blue curve. The optimal cover thickness along this curve is found to be $H_{\mathrm{pd}}=80\;µm$ at f=2.070 MHz, identified as resonance mode “A”, which yields an acoustic energy density of 96 J/m${}^{3}$, twenty times larger than “A${}^{\prime }$”.

In Figure 5b–d are shown experimental and simulated acoustophoretic particle velocities $v_{\mathrm{pa}}$ for three different particle diameters 2a=4.8, 2.0 and 1.0 µm in mode “A${}^{\prime }$”. The magnitude $v_{\mathrm{pa}}=38\;µm/s$ for a=2.4µm in Figure 5b is similar to that for mode “S”, $v_{\mathrm{pa}}=40\;µm/s$ in Figure 3c. As for mode “S” in Figure 3, also Figure 5 shows the well-known cross-over from radiation-force-dominated focusing motion of the largest particles to the streaming-roll-dominated motion of the smallest particles. Particular to the single-PDMS-cover device is, firstly, that in Figure 3c and Figure 5d there is only one pair of counter-rotating vortices near the bottom aluminum wall, the other pair near the top-PDMS-cover is suppressed as anticipated, and secondly, very strong localized vortices appear in the top corners of the channel where the PDMS-cover joins the aluminum base.

For both mode “S” in Figure 3 and the anti-symmetric mode “A${}^{\prime }$” in Figure 5b–d there is good qualitative agreement between particle velocities $v_{\mathrm{pa}}$ and resonance frequencies *f* measured in 3D and simulated in 2D assuming translational invariance. Quantitatively, the agreement in *f* is better for mode “A${}^{\prime }$” (+2%) than for mode “S” (−12%), presumably because the translational invariance is more severely broken in the latter case. In both cases the velocity magnitudes agrees within 30–50%. The skew (white) zero-velocity band observed in Figure 5b (left) indicates that the experimental device was not completely symmetric as in the model Figure 5b (right). The agreement between measured and simulated particle velocities $v_{\mathrm{pa}}$ implies that our model can provide reliable estimates of the acoustic energy densities $E_{\mathrm{ac}}$ in the devices.

### 4.2. The Role of Variations in the PDMS Material Properties

**Figure 6:**
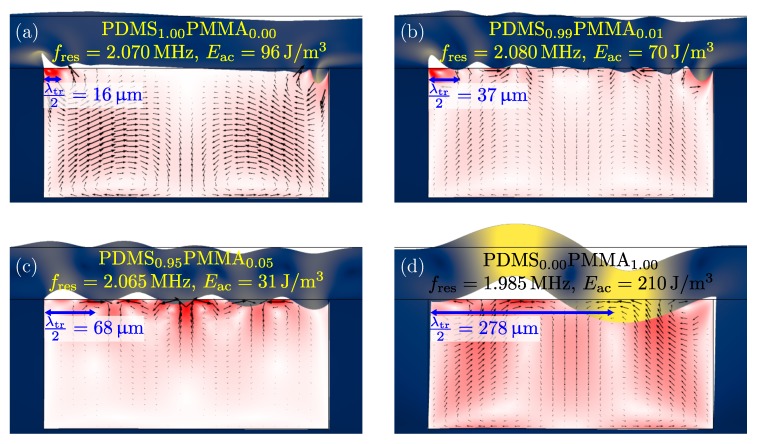
Zoom-in on the resonance modes for four different mixing ratios *x* of the artificial polymer alloy PDMS${}_{x}$PMMA${}_{1-x}$ with (**a**) x=1.00, (**b**) x=0.99, (**c**) x=0.95, and (**d**) x=0.0. For each case, the resonance frequency $f_{\mathrm{res}}$ is noted together with the average acoustic energy density $E_{\mathrm{ac}}$, and the transverse wavelength $\lambda_{\mathrm{tr}}$ in the artificial PDMS-PMMA polymer cover. The color plots indicates the particle velocity magnitude $|v_{\mathrm{pa}}|$ in the water ranging from 0 (white) to the maximum value $v_{\mathrm{pa}}^{\max}=258\;µm/s$ (red), and of the displacement u in surrounding solids ranging from 0 (dark blue) to 70 nm (yellow). The deformation is scaled 500 times to be visible.

We suspect that the strong top-corner vortices appearing in Figure 3 and Figure 5 are due to the nearly incompressible nature of PDMS, or equivalently, the very low transverse speed of sound in PDMS. To investigate this hypothesis, we define an artificial polymer alloy of PDMS and Poly(Methyl MethAcrylate), PDMS${}_{x}$PMMA${}_{1-x}$ with mixing ratios *x*. In Figure 6 we study the four mixing ratios x=1.0, 0.99, 0.95, and 0 in covers 80µm thick. For each case, the resonance peak with the largest figure of merit F was found, and in close-up views of the channel are shown the corresponding particle velocity $v_{\mathrm{pa}}$ in the water and the displacement u in the surrounding solid.

In the case of a pure PDMS cover Figure 6a, a large displacement is narrowly confined to the top corners, this was also evident in Figure 3 and Figure 5, where the maximum particle velocity was seen near the top corners. Already at 99% PDMS and 1% PMMA, Figure 6b, the transverse speed of sound in the polymer is two times greater than in pure PDMS, and the displacement field at the polymer-water interface is now more evenly distributed. This is even more pronounced for the case of 95% PDMS and 5% PMMA, Figure 6c, similarly for pure PMMA, Figure 6d. Note that in the latter case, the displacement in the cover is also resonating close to its transverse half-wave frequency. In conclusion, the low transverse speed of sound in PDMS seems to imply the appearance of strong streaming vortices localized near the PDMS-aluminum corners of the device.

## 5. Modeling Dual-PDMS-Cover Aluminum Devices with Anti-symmetric Voltage Actuation

**Figure 7:**
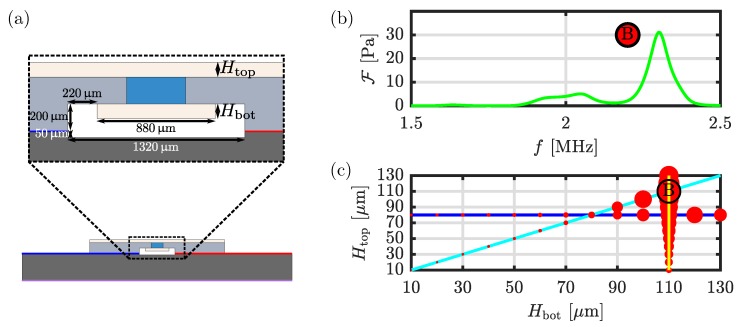
(**a**) Zoom-in on the channel in the dual-PDMS-cover setup, indicating the top cover thickness $H_{\mathrm{top}}$ and the bottom cover thickness $H_{\mathrm{bot}}$. (**b**) Plot of the focusing figure of merit F versus frequency computed for the case $H_{\mathrm{top}}=H_{\mathrm{bot}}=110\;µm$ leading to the identification of a strong resonance peak “B” at f=2.302 MHz. (**c**) Scatter plot with area of the points proportional to the focusing figure of merit F for the strongest resonances identified in parametric sweeps of $H_{\mathrm{bot}}$ for fixed $H_{\mathrm{top}}=80\;µm$ (blue line), of $H_{\mathrm{top}}$ for fixed $H_{\mathrm{bot}}=110\;µm$ (yellow line), and for equal cover thicknesses $H_{\mathrm{top}}=H_{\mathrm{bot}}$ (cyan line). The configuration with the highest focusing quality is indicated with an encircled B.

In Figure 3c,d and Figure 5d showing the acoustophoretic velocity fields for small microparticles suspended in the single-PDMS-cover device, we notice that whereas the usual Rayleigh flow rolls are present near the bottom aluminum wall, they seem to be suppressed near the PDMS cover. We therefore study the dual-PDMS-cover device sketched in Figure 7a to examine if both sets of Rayleigh flow rolls near the top and bottom wall can be suppressed. In Figure 7b we show one example of plotting the focusing figure of merit F of Equation (15) as a function of frequency *f*, here for the specific case of $H_{\mathrm{top}}=H_{\mathrm{bot}}=110\;\mathrm{µm}$. The strongest resonance is found at f=2.302 MHz and is marked “B”. A point with an area proportional to the computed value of F is then plotted at $\left(H_{\mathrm{top}},H_{\mathrm{bot}}\right)=\left(110\;\mathrm{µm},110\;\mathrm{µm}\right)$ in the scatter plot of Figure 7c. Similarly, the strongest resonances are found and plotted in the scatter plot for three different parametric sweeps in the two cover thicknesses: (1) $H_{\mathrm{bot}}$ varies for fixed $H_{\mathrm{top}}=80\;µm$ (blue line), (2) $H_{\mathrm{top}}$ varies for fixed $H_{\mathrm{bot}}=110\;µm$ (yellow line), (3) the equal cover thicknesses $H_{\mathrm{top}}=H_{\mathrm{bot}}$ varies (cyan line). The strongest resonance or best value of the focusing figure of merit F is found at the peak “B” corresponding to the equal cover thicknesses $H_{\mathrm{top}}=H_{\mathrm{bot}}=110\;µm$ driven at the frequency f=2.302 MHz.

**Figure 8:**
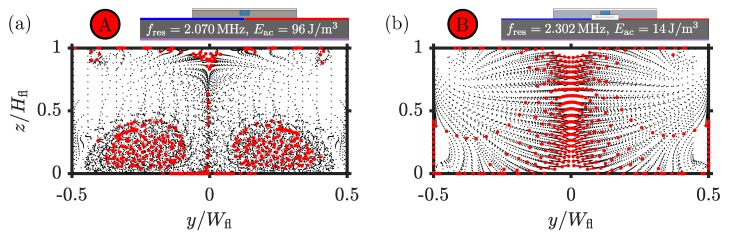
The positions (black dots) of suspended 1-µm-diameter polystyrene particles at discrete time steps from initial time t=0 to final time $t=t_{\mathrm{focus}}$ in anti-symmetrically actuated devices at selected resonance modes. The final positions at time $t=t_{\mathrm{focus}}$ are marked by red dots. (**a**) Resonance mode “A” of Figure 5 with $E_{\mathrm{ac}}=96\;J/m^{3}$ and $t_{\mathrm{focus}}=4.3$ s with two large vortices extending from the hard bottom aluminum-water interface, two small ones near the PDMS-aluminum corners and poor focusing. (**b**) Resonance mode “B” of Figure 7 with $E_{\mathrm{ac}}=14\;J/m^{3}$ and $t_{\mathrm{focus}}=23.6$ s exhibiting four small vortices near the corners and good focusing.

In Figure 8, we study if indeed the streaming flow rolls are suppressed in the dual-PDMS-cover device compared to the single-PDMS-cover device. We compute the position of 400 1-µm-diameter polystyrene particles at discrete time steps (black dots) starting at a uniform distribution at time t=0, where the acoustic field is turned on, and ending at time $t_{\mathrm{focus}}=\frac{3}{2\Phi }\frac{c_{\mathrm{fl}}^{2}}{\omega^{2}a^{2}}\frac{\eta_{\mathrm{fl}}}{E_{\mathrm{ac}}}$(red dots), the so-called focusing time determined by the acoustic contrast factor Φ given in Table A3 in Appendix A. Inertial effects can be ignored since the Reynolds number Repa=ρflavpaηfl<0.03 for the given particles and acoustic fields.

Comparing the particle trajectories of mode “A” in the single-PDMS-cover device Figure 8a to those of mode “B” in the dual-PDMS-cover device Figure 8b, we clearly see that in the latter case the streaming flow rolls are suppressed. The dual-PDMS-cover device is therefore predicted to be a good candidate system for controlled focusing of sub-micrometer-sized particles. For this to work, it is of course essential that the focusing time is sufficiently short. For the given device actuated at $V_{0}=10\;V$, the focusing time of the 1-µm-diameter particles is prohibitively long, namely $t_{\mathrm{focus}}=23.6$ s. Raising the driving voltage by a factor $\sqrt{20}\approx 4.5$ to $V_{0}=45\;V$ would lower the focusing time to $t_{\mathrm{focus}}\approx 1$ s, because $t_{\mathrm{focus}}\propto V_{0}^{2}$.

## 6. Conclusions

We have developed a model for analyzing the single-PDMS-cover aluminum-base device with side actuation, recently introduced by Gautam et al. [20]. The model, currently restricted to the case of a constant 2D cross section in a translational invariant device, is validated experimentally with fair qualitative and quantitative agreement by fabricating and characterizing two types of single-PDMS-cover aluminum-base devices: One which is actuated with a symmetric ac-voltage on a Pz26 transducer placed at the side of the channel, and another with an anti-symmetric ac-voltage on a transducer placed right under the channel. Both numerical simulations and experiments support our hypothesis that using a soft PDMS cover of the acoustophoresis channel, the boundary driven acoustic streaming is suppressed in the bulk. The developed model can thus predict the streaming patterns in such devices, and we subsequently used it to show three aspects: (1) The incompressible nature of the soft PDMS cover introduces strong streaming rolls confined near the corners where the PDMS cover joins the aluminum base, while maintaining the conventional large Rayleigh streaming rolls extending from the aluminum-water interface; (2) An optimal thickness of the PDMS cover can be determined by simulation; (3) In devices with a dual-PDMS cover, the model predicts that the conventional Rayleigh streaming flow rolls should be suppressed and changed into vortices confined near the corners of the channel. Experimental work is in progress to verify these predictions.

## Figures and Tables

**Table 1:** Dimensions of the two chip geometries shown in Section 1 and Section 4.

Chip		Units	Pz26	Electrodes	Al (Main)	Al (Side)	PDMS	Channel
	Length	mm	25	25	60	30	60	44
Section 1	Width	mm	5	5	5	4	5	0.44
	Height	mm	1	0.009	0.4	0.4	1.5	0.2
	Length	mm	25	25	60	–	60	44
Section 4	Width	mm	10	10	5	–	5	0.44
	Height	mm	1	0.009	0.4	–	1.5	0.2

## Appendix A. Material Parameters

The following three tables contain the parameter values used in the numerical modeling of the PDMS-cover aluminum-based devices.

**Table A1:** Parameters for PZT transducer Pz26 [27] with damping coefficient $\Gamma_{\mathrm{sl}}=0.02$ [28].

Parameter	Value		Parameter	Value		Parameter	Value	
$\rho_{\mathrm{sl}}$	7700	kg/m${}^{3}$	$\varepsilon_{11}$	828	$\varepsilon_{0}$	$\varepsilon_{33}$	700	$\varepsilon_{0}$
$C_{11}$	168	GPa	$C_{33}$	123	GPa	$e_{31}$	−2.8	C/m${}^{2}$
$C_{12}$	110	GPa	$C_{44}$	30.1	GPa	$e_{33}$	14.7	C/m${}^{2}$
$C_{13}$	99.9	GPa	$C_{66}$	29.0	GPa	$e_{15}$	9.86	C/m${}^{2}$

**Table A2:** Parameters used in the linear-elastics modeling of aluminum, silver, and PDMS.

		Aluminum	Silver	PDMS (10:1)	
Parameter	Symbol	6061 [29]	[30]	Cured at 65 ${}^{\circ }$C [31]	Unit
Mass density	$\rho_{\mathrm{sl}}$	2700	10,485	1029	kg/m${}^{3}$
Elastic modulus	$C_{11}$	$102\left(1-i\Gamma_{\mathrm{sl}}\right)$	$134\left(1-i\Gamma_{\mathrm{sl}}\right)$	1.08−i0.016	GPa
Elastic modulus	$C_{44}$	$25.9\left(1-i\Gamma_{\mathrm{sl}}\right)$	$25.9\left(1-i\Gamma_{\mathrm{sl}}\right)$	0.0075−i0.0079	GPa
Damping coefficient	$\Gamma_{\mathrm{sl}}$	0.0013	0.0004	–	–

**Table A3:** Parameters for water and polystyrene tracer particles at 25 ${}^{\circ }$C. The scattering coefficients are calculated in terms of the mass densities and compressibilities as $f_{0}=1-\frac{\kappa_{\mathrm{pa}}}{\kappa_{\mathrm{fl}}}$ and $f_{1}=\frac{2\left(\frac{\rho_{\mathrm{pa}}}{\rho_{\mathrm{fl}}}-1\right)}{2\frac{\rho_{\mathrm{pa}}}{\rho_{\mathrm{fl}}}+1}$ which in turn gives the acoustic contrast factor $\Phi =\frac{1}{3}f_{0}+\frac{1}{2}f_{1}$.

Water [32]			Polystyrene Particles [33]		
Parameter	Symbol	Value	Parameter	Symbol	Value
Mass density	$\rho_{\mathrm{fl}}$	997.05 kg/m${}^{3}$	Mass density	$\rho_{\mathrm{pa}}$	1050 kg/m${}^{3}$
Compressibility	$\kappa_{\mathrm{fl}}$	447.7 TPa${}^{-1}$	Compressibility	$\kappa_{\mathrm{pa}}$	238 TPa${}^{-1}$
Dynamic viscosity	$\eta_{\mathrm{fl}}$	0.890 mPa·s	Monopole coefficient	$f_{0}$	0.468
Bulk viscosity	$\eta_{\mathrm{fl}}^{b}$	2.485 mPa·s	Dipole coefficient	$f_{1}$	0.034
Speed of sound	$c_{\mathrm{fl}}$	1496.7 m/s	Contrast factor	Φ	0.173
